# The genomic origin of Zana of Abkhazia

**DOI:** 10.1002/ggn2.10051

**Published:** 2021-06-14

**Authors:** Ashot Margaryan, Mikkel‐Holger S. Sinding, Christian Carøe, Vladimir Yamshchikov, Igor Burtsev, M. Thomas P. Gilbert

**Affiliations:** ^1^ Section for Evolutionary Genomics, GLOBE Institute Faculty of Health and Medical Sciences University of Copenhagen Copenhagen Denmark; ^2^ Center for Evolutionary Hologenomics University of Copenhagen Copenhagen Denmark; ^3^ Smurfit Institute of Genetics Trinity College Dublin Dublin Ireland; ^4^ Division of Drug Discovery Southern Research Institute Birmingham Alabama USA; ^5^ International Center of Hominology State Darwin Museum Moscow Russia; ^6^ Department of Natural History NTNU Trondheim Norway

**Keywords:** Abkhazia, Almasty myth, ancient DNA, Caucasus, East Africa, enslavement, human migration, human trafficking

## Abstract

Enigmatic phenomena have sparked the imagination of people around the globe into creating folkloric creatures. One prime example is Zana of Abkhazia (South Caucasus), a well‐documented 19th century female who was captured living wild in the forest. Zana's appearance was sufficiently unusual, that she was referred to by locals as an Almasty—the analog of Bigfoot in the Caucasus. Although the exact location of Zana's burial site was unknown, the grave of her son, Khwit, was identified in 1971. The genomes of Khwit and the alleged Zana skeleton were sequenced to an average depth of ca. 3× using ancient DNA techniques. The identical mtDNA and parent‐offspring relationship between the two indicated that the unknown woman was indeed Zana. Population genomic analyses demonstrated that Zana's immediate genetic ancestry can likely be traced to present‐day East‐African populations. We speculate that Zana might have had a genetic disorder such as congenital generalized hypertrichosis which could partially explain her strange behavior, lack of speech, and long body hair. Our findings elucidate Zana's unfortunate story and provide a clear example of how prejudices of the time led to notions of cryptic hominids that are still held and transmitted by some today.

## Graphical abstract

The displaced origins of a misconstrued person. Face reconstruction from Tkhina‐75 skull. Bottom right: artistic reconstruction after consideration of contemporary witness accounts.

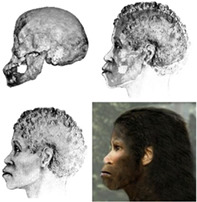

## 1 INTRODUCTION

The local folklore of the South Caucasus region of Abkhazia records a “wild woman” named Zana, who lived in the 19th century, who was referred to by some locals as a female Abnauayu or Almasty: names for a creature similar to the infamous Yeti of the Himalayas and Bigfoot of North America, that supposedly lives in the Caucasus and Central Asia.^1^, ^2^ Originally captured while living outdoors in the forest, Zana was later enslaved by a succession of local wealthy individuals, and was finally bought by the Abkhaz nobleman Edgi Genaba who took her to his estate at Tkhina, where she lived until her death around 1890.^3^

Inspired by the speculation that she might have been a female Yeti, Soviet scientists visited the region in 1962 to gather descriptions and accounts from the elders living in the village of Tkhina, who still recalled her. The locals described her as being “part human and part animal,” 2 m tall and dark‐skinned, covered with thick hair, who was able to lift a 50 kg sack of flour with one hand, and outrun a horse in a race.^1^, ^3^ According to the eyewitness accounts she also lacked speech, which along with her alleged strange behavior and appearance, likely resulted in her reputation as an Almasty. Zana is also documented to have given birth to two sons and two daughters from local men. Following her death, she was buried in the Genabas' family cemetery, and although the exact location of Zana's burial site was unknown, the grave of her youngest son, Khwit, was identified in 1971. After several attempts to locate Zana's burial site, the remains of an anonymous female were discovered in the Genaba's family cemetery, leading to speculation that they may have belonged to Zana herself.^4^, ^5^

To overcome limitations of a previous DNA analysis and the possible ambiguities of craniometric studies, we sequenced the genomes of both the unknown female and Khwit, to 3.1‐ and 3.3‐fold coverage, respectively. We performed genome‐wide analysis to explore their genetic ancestry and kinship relations, which allowed us to shed light on Zana's story based on objective genome‐wide data.

## 2 MATERIAL AND METHODS

See the Supplementary Methods for a more in‐depth description of the materials and methods used in this study.

### 2.1 Data generation and bioinformatics analysis

All ancient DNA (aDNA) laboratory work was carried out in dedicated clean laboratory facilities at the GLOBE Institute, University of Copenhagen, according to aDNA standards described elsewhere.^6^

We used teeth and petrous bones to extract ancient DNA from both individuals and build double‐stranded BGISeq libraries following the BEST protocol, using adapters compatible with BGI sequencing according to Mak et al. 2017.^7^ The amplified libraries were sequenced on two lanes of BGISEQ‐500 platform.

We used the BAM workflow implemented in the PALEOMIX pipeline^8^ to trim and map the sequencing reads against the human reference genome build GRCh37 and the revised Cambridge reference sequence (rCRS, NCBI accession number NC_012920.1).

We used mapDamage v2.0 to get the read length distribution and approximate Bayesian estimates of damage parameters.^9^ To estimate the levels of contamination in the ancient samples we used contamMix^10^ and the X‐chromosome based contamination method implemented in ANGSD.^11^ The sex of individuals were assigned according to the Rγ estimates described elsewhere.^12^

### 2.2 Uniparental marker and kinship analyses

We used haplogrep2^13^ for mtDNA haplogroup assignment. For determining Khwit's Y chromosome haplogroup we used the pathPhynder workflow (https://github.com/ruidlpm/pathPhynder).

A phylogenetic network analysis of complete mtDNA sequences including that of “Zana”, Khwit and other L2 haplogroup sequences^14^ (n = 93) was conducted with POPART^15^ using the “Median Joining Network” algorithm. We used BEAST v2.6.1^16^ to estimate the divergence time of “Zana's” mtDNA lineage using the Bayesian skyline plot (BSP) method. The tree height was calibrated based on the previous work by Silva et al.^14^

The kinship coefficients were calculated by first generating a site allele frequency likelihood file (saf) in ANGSD (http://www.popgen.dk/software/index.php/IBSrelate) which was followed by estimation of IBS sharing matrix based on the two‐dimensional site frequency spectrum (2d‐SFS) from real‐SFS implemented in ANGSD.^11^

### 2.3 Population genetics

To assess the genetic relationship between “Zana,” Khwit, and other populations, we merged the shotgun sequencing data from the historical individuals with the Affymetrix Human Origins SNP array panel of worldwide populations.^17^, ^18^ We also included data from three archaic humans (two Neanderthals^19^ and the Denisovan^20^) and chimpanzee genotypes, as well as genomes from two Caucasus hunter‐gatherer (CHG) individuals (the ca. 13300 years old SATP specimen, and the ca. 9700 years old KK1 specimen ) originally excavated in the South Caucasus^21^ for comparison.

We conducted unsupervised maximum likelihood‐based clustering analysis with ADMIXTURE^22^ by pruning the data set for linkage disequilibrium using plink v1.9.^23^ The program pong^24^ was used to identify and visualize the best run for each K and similar components between different Ks.

The principal components analysis (PCA) was performed using plink v1.9 with the ancient genomes projected onto the modern variation. The first 30 eigenvectors of PCA were used as input for the uniform manifold approximation and projection (UMAP) analysis using the “umap” R package.

D‐statistics estimates were calculated using the ADMIXTOOLS^25^ and R package “admixr.”^26^ The maximum likelihood phylogenetic tree of “Zana” and the African populations were constructed with TreeMix.^27^

## 3 RESULTS AND DISCUSSION

A total of 1 219 599 801 BGISeq sequences were generated from the teeth and petrous bones for both individuals (see Mapping Statistics in Table S1). As expected from the results of previous studies,^28^ the endogenous content was much higher in the petrous bones (“Zana”—41.95%, Khwit—33.93%) than teeth samples (“Zana”—1.16%, Khwit—12.7%). We used the data to obtain the genomes at an average sequencing depth of coverage of 3.1× and 3.3×, for “Zana” and Khwit, respectively. The sequences show typical ancient DNA damage profiles and short DNA fragment lengths,^29^ even though the individuals died relatively recently; Zana lived until around 1890 and Khwit until 1954.

The contamination estimates based on mtDNA (for both) and X chromosome (only for Khwit) were less than 1%, and the chromosomal sexes of the individuals matched their anthropological descriptions: Khwit as male and “Zana” as female.

### 3.1 Uniparental markers

The mtDNA sequences were identical for both individuals, consistent with the hypothetical mother‐son relationship, and could be assigned to haplogroup L2b1b, the parental haplogroup of which (L2b clade) is widely distributed in western Africa,^14^, ^30^ but is also found across Africa.^31^ The same haplogroup was identified by an earlier, independent analysis of teeth samples from Khwit and “Zana” at the Southern Research Institute based on human DNA enriched libraries (unpublished data).

Khwit's Y‐chromosomal lineage belongs to the haplogroup R1b1a1b1b which clearly reflects his non‐African paternal heritage. This is a sub‐haplogroup of a major haplogroup R1b1a1b defined by M269 mutation, which is encountered in high frequencies in Europe and western Asia.^32^, ^33^, ^34^

**FIGURE 1:**
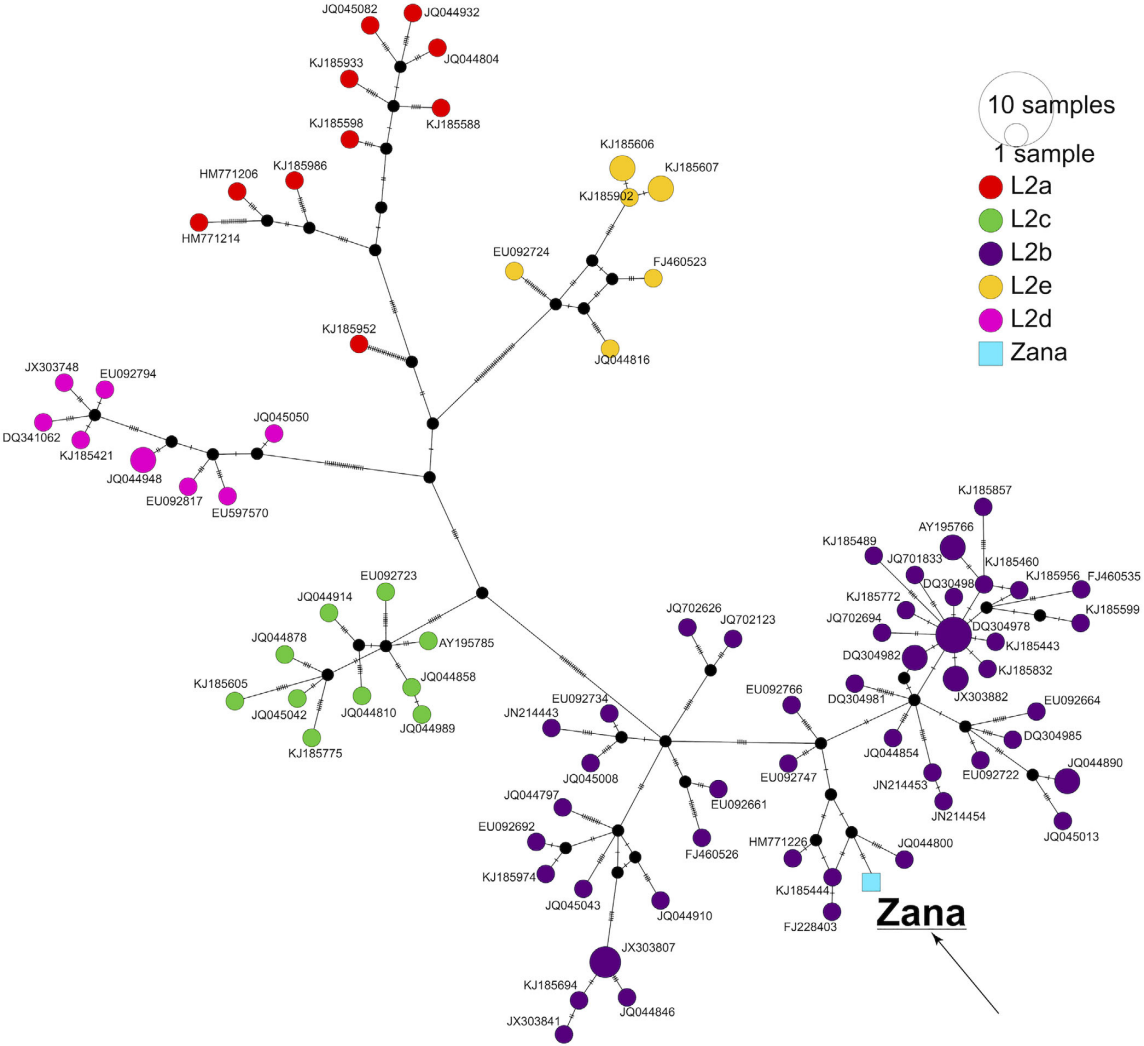
Median‐joining network. The analysis of mtDNA sequences of “Zana” and Khwit alongside 93 complete mtDNA sequences from the human L2 mitochondrial clade using the “Median‐Joining” algorithm implemented in PopArt. Each circle represents a certain haplotype; smaller black circles indicate median vectors. Small black lines connecting branches between the haplotypes denote the number of mutation steps separating the haplotypes. Since the mtDNA sequences of “Zana” and Khwit are identical, only Zana's mtDNA haplotype is mentioned in the plot

Although an earlier study that analyzed Khwit's mtDNA sequence revealed "Zana's" maternal origin as African,^35^ the authors suggested that "Zana" could have belonged to an ancient African lineage, likely due to the lack of a suitable comprehensive comparative data set at that time. We therefore, conducted a mitochondrial network analysis to assess the relationship of the putative “Zana” sample's maternal lineage with other (n = 93) human L2 haplogroup sequences,^14^ and found that it clusters together with other individuals of the L2b lineage, as expected (Figure 1).

Silva et al. have estimated that mitochondrial clade L2b likely originated ca. 24 kya,^14^ thus our mtDNA assignment can be used to reject the hypothesis that the hypothetical “Zana” sample had an ancient or archaic origin. In order to obtain an approximate estimate of the time to the most recent common ancestor (TMRCA) of the maternal lineage of “Zana” and its sister groups, we ran BEAST based only on L2b sequences (n = 57) (Figure S3). With the limited number of L2b1b sequences (n = 4) in the data set, the divergence time was estimated to be ca. 9800 ya (3515‐13 000; 95% highest posterior density intervals).

### 3.2 Kinship

We next further tested whether the two individuals were directly related using PC‐relate as implemented in PCAngsd^36^ and realSFS.^11^ A kinship coefficient of 0.1818 was obtained for the pair of “Zana” and Khwit, thus falling within the first degree relationship interval (0.177‐0.354) as described in Manichaikul et al.^37^ An R0 value (R0 = 0.0015) close to 0 was estimated based on the realSFS analysis, suggesting a parent‐offspring relationship, which together with the identical mtDNAs indicate that the unknown female skeleton was Khwit's mother, thus can be identified positively as Zana.

### 3.3 Genetic Affinities

To further assess the genetic relationship between Zana, Khwit and various worldwide populations using nuclear genome variation, we ran a PCA based on the Human Origins (HO) panel. To aid visualisation, we reduced the total number of reference populations in the panel to represent the major genetic lineages of the world. However, given its geographic relevance, we included relatively more populations from the Caucasus, and given previous hypotheses that Zana may have had some archaic hominid ancestry, we also included genome‐wide data from three archaic humans, and used the chimpanzee as an outgroup. Additionally, we included the two Mesolithic hunter‐gatherers (SATP and KK1) from the South Caucasus for comparison.

**FIGURE 2:**
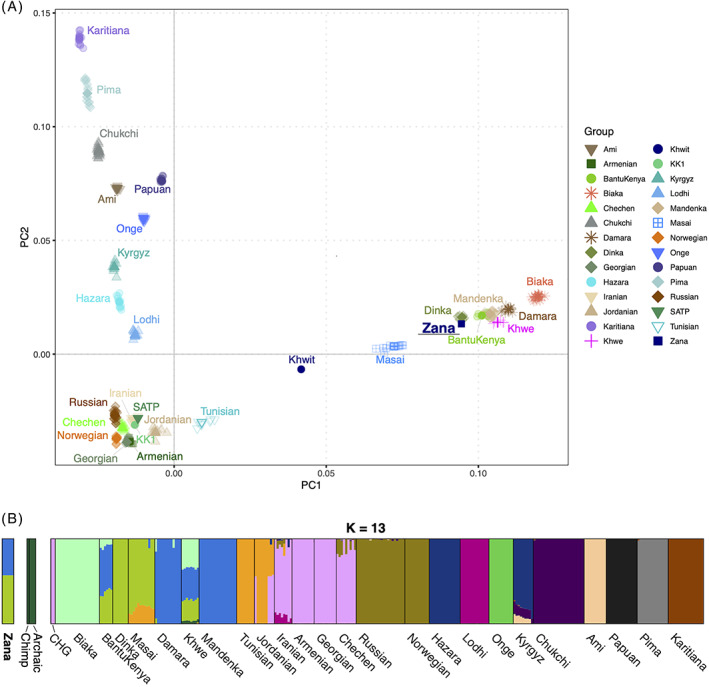
PCA and admixture analyses. A, PCA analysis of Zana, Khwit, HO panel of worldwide present‐day populations and two CHG (KK1 and SATP) samples. The four ancient genomes were projected onto the modern variation. B, Model‐based clustering analysis using ADMIXTURE, (K = 13) to estimate Zana's ancestry proportions. A total of 300 individuals with 190 111 SNPs were used for the analysis

The results clearly show that Zana is neither genetically close to archaic humans nor the chimpanzee, but clusters closely with modern human populations (Figure 2A). As expected from kinship (parent‐offspring) and Y‐chromosome (European R1b1a1b lineage) analyses, Khwit has an intermediate location on the PCA plot between European or Caucasian and African populations.

**FIGURE 3:**
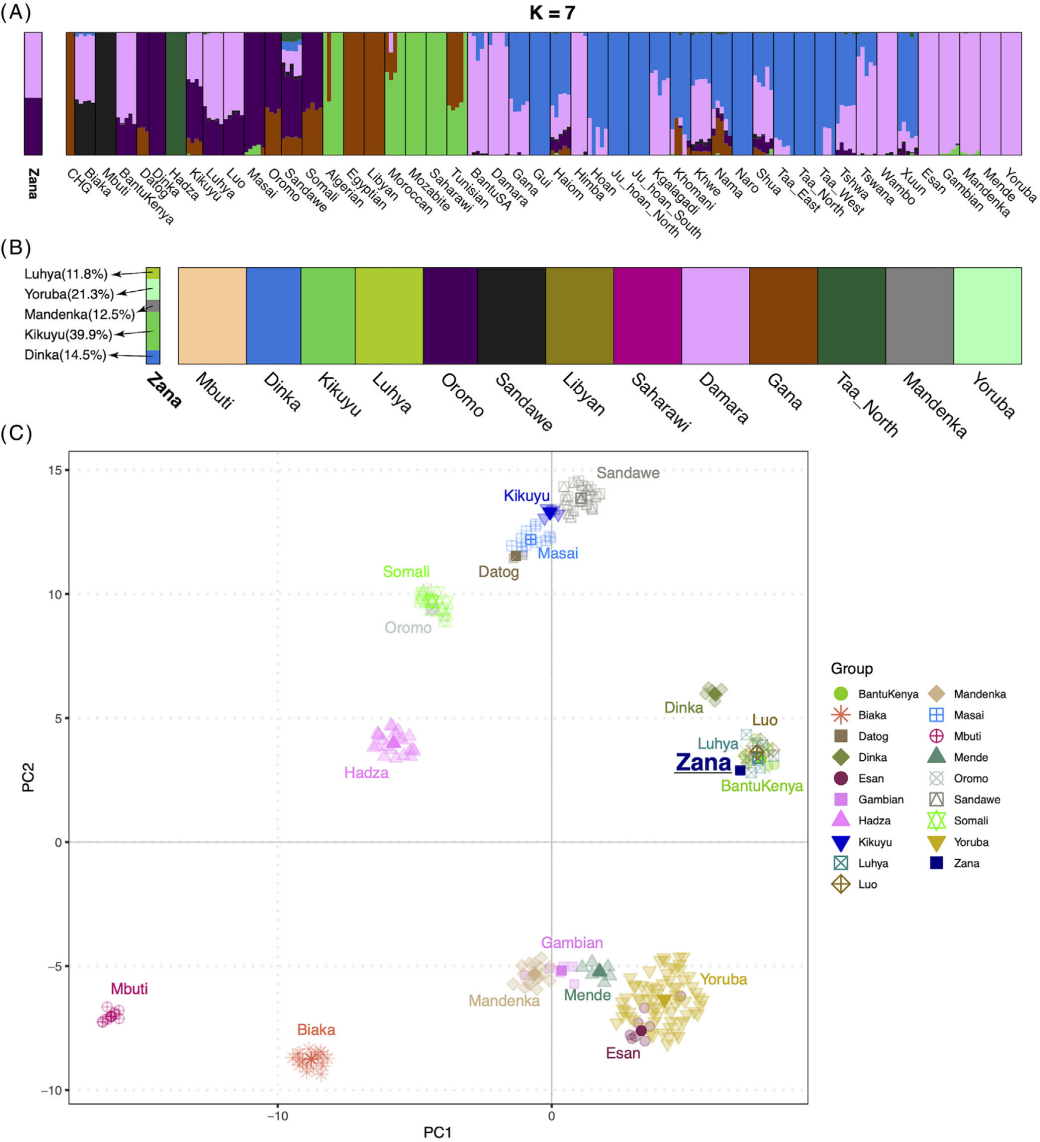
Supervised/unsupervised admixture and PCA analyses (A, B, and C, will be added to the plot). A, Model‐based clustering analysis using ADMIXTURE, (K = 7) using ~approximately five individuals from each of 48 African groups (n = 232) and 270 311 markers. B, Zana's genetic ancestry proportions using K = 13 African potential source populations using the “supervised mode” implemented in ADMIXTURE. The results are based on 300 replicates with different seed values. C, UMAP analysis of Zana along with eastern, central, and western African groups

Unsupervised clustering analysis using ADMIXTURE also clearly rejects any hypothesis that Zana was of “nonhuman” origin, for example as suggested by various sources.^1^, ^2^ Rather, it is clear that she shared genetic ancestry with present‐day western and eastern African populations (Figure 2B). To explore this African origin further, we conducted additional PCA and admixture analyses based solely on African groups from the HO panel (Figure 3A and Figure S4). Here again, Zana shows ancestry components from the eastern (eg, Dinka) and western (eg, Yoruba) African groups, with no significant genetic contribution from southern, northern, and central African populations. We were unable, however, to resolve whether she was (a) an individual derived from admixture between a Dinka‐like and Yoruba‐like population (purple and plink components in Figure 3A) or (b) originated solely from eastern African groups such as Luhya and Luo.

**FIGURE 4:**
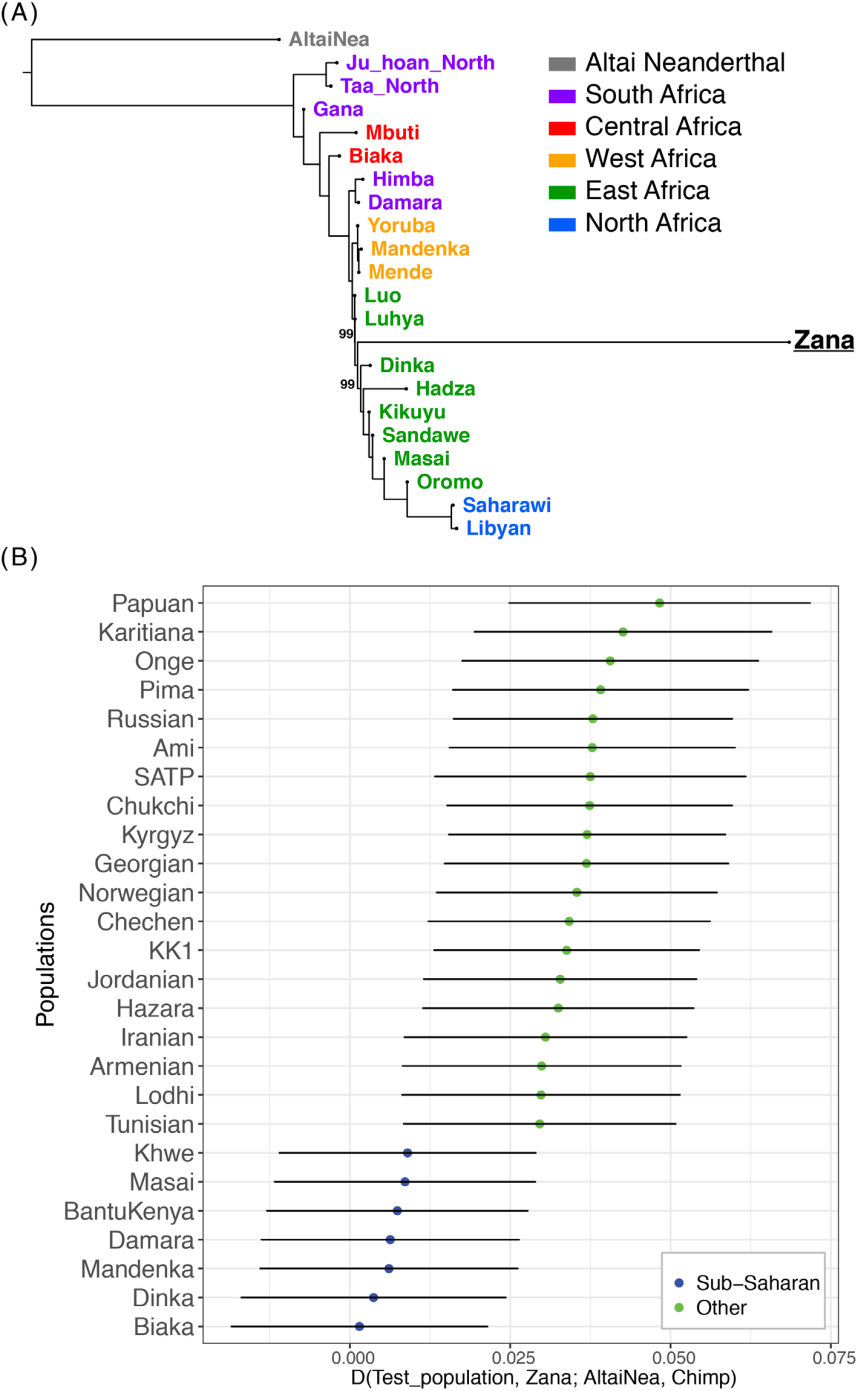
Maximum likelihood phylogenetic tree and *D*‐statistics. A, TreeMix analysis of Zana and African populations from the HO panel. This represents the consensus tree based on 100 replicates with random seed values. A total of 98 individuals from 22 populations and 101 799 transversion sites (to reduce aDNA‐related biases in Zana's genome) were used for the analysis. We used a reduced number of populations to aid visualisation. The excess drift signal is an artifact of the pseudo‐haploid nature of the reconstructed genome of Zana. B, 𝐷‐statistics of the form (Test_population, Zana; AltaiNea, Chimpanzee) suggesting that there is no significant increase in Neanderthal admixture in Zana compared with Sub‐Saharan populations (Z < 2), while the rest of the world‐wide population demonstrate the well‐known Neanderthal admixture (Z > 2). The bars represent ±2 SE estimates

To estimate Zana's ancestry proportion more accurately, we conducted the admixture analysis in “supervised” mode based on 13 African populations (Figure 3B) representing most of the diversity of human populations in Africa. Even though the results confirm Zana's largely eastern (~66%) African origin, she also displayed significant levels of western African (~34%) genetic component. To further visualise Zana's genetic relationship with the African populations, we applied additional dimensionality reduction using UMAP with only eastern, central, and western African groups, which reveals Zana's genetic proximity with eastern African populations such as Luo and Luhya (Figure 3C). This is also supported by the maximum likelihood analysis based on TreeMix (Figure 4A).

To formally test the possible Neanderthal introgression into Zana's genome we conducted 𝐷‐statistics in the form of (Test_population, Zana; AltaiNea, Chimpanzee) (Figure 4B). The results indicate Neanderthal admixture only for populations of northern African and non‐African populations (well‐known from previous studies^19^, ^38^) which is in line with Zana's genetic proximity with sub‐Saharan groups identified based on the clustering analyses.

In summary, our results based on genome‐wide analysis reveal that Zana's genome had a sub‐Saharan African genetic origin, consistent with the results of previous (unpublished) craniometric and mtDNA analyses. This suggests that her presence in the region may have been linked to the Ottoman Empire slave trade to Istanbul, that was one of the main hubs for the slave trade in the region in the 19th century. Moreover, Zana's largely eastern African ancestry is consistent with the historical records indicating that most of the African slaves in the Ottoman Empire originated around areas of the African Great Lakes and present‐day Sudan.

The contemporary reports and subsequent tales of Zana's wildness were at least partially based on some of her unusual physical characteristics such as the lack of speech, intellectual disability and long hair covering her whole body to name a few. With the genomic data clearly rejecting all nonhuman hypotheses, we speculate that if these descriptions of her physical characteristics are accurate, she may have had a rare human genetic disorder such as congenital generalised hypertrichosis: a syndrome with dismorphic facial features, intellectual disability, and hypertrichosis.^39^

## 4 CONCLUSIONS

Our results prove that the unknown female buried in the Genaba family cemetery was Zana herself. In contrast to the speculations that she might have been a female Almasty, we provide definitive genome‐wide data to put an end to the accounts of her as anything but a human woman.

Zana was likely of eastern African descent, although we cannot rule out partial western African ancestry. We hypothesise that her lineage could have arrived in the territory of present‐day Abkhazia (South Caucasus) as a result of the slave‐trade practiced between the 16 to 19th centuries CE by the Ottoman Empire. Lastly, we speculate that it was simply her unfamiliar individual physical characteristics (such as unusual behavior, physical strength, tall stature, lack of recognisable speech and hypertrichosis) and the subsequent rumors over generations that fueled the myth of a non‐human origin.

### 4.1 A note on ethics

Following her capture in the forest, Zana was deprived of her basic human rights, and treated as a slave: she was kept in captivity, likely forced to have sexual relations with local men, and worked in forced labor conditions. After she passed away, the accounts on her mythical figure attracted several scientists to unearth her story and her son's bones were exhumed. Our study intends, both to reveal the true human nature of Zana and grant her and her descendants' remains the dignity they deserve.

All permissions for excavations in the 1960–1970s and for aDNA analyses were provided by the relevant authorities.

## AUTHOR CONTRIBUTIONS

**Margaryan, Ashot:** Conceptualization; Data curation; Formal analysis; Methodology; Project administration; Supervision; Validation; Visualization; Writing‐original draft; Writing‐review & editing. **Mikkel‐Holger Sinding:** Data curation; methodology; validation; writing‐review & editing. **Christian Carøe:** Data curation; investigation; methodology; validation; writing‐review & editing. **Vladimir Yamshchikov:** Conceptualization; data curation; methodology; validation; writing‐review & editing. **Igor Burtsev:** Conceptualization; investigation; methodology; validation; writing‐review & editing. **M. Thomas Gilbert:** Conceptualization; funding acquisition; investigation; project administration; supervision; writing‐original draft; writing‐review & editing.

## CONFLICT OF INTEREST

The authors declare no competing interests.

### PEER REVIEW

The peer review history for this article is available at https://publons.com/publon/10.1002/ggn2.10051 and in Supplementary File 3.

## Supporting information


**Appendix** **S1:** Supplementary Note
**Supplementary Figure S1**.1 Zana's descendants

Supplementary Table 1

Supplementary TPR File

## Data Availability

The data that support the findings of this study are openly available in European Nucleotide Archive (ENA) at https://www.ebi.ac.uk/ena/, reference number PRJEB45032.
