# Supplementary material for: The genomic origin of Zana of Abkhazia
Source: Adv Genet (Hoboken). 2021 Jun 14;2(2):e10051. doi: 10.1002/ggn2.10051 (PMC9744565; doi:10.1002/ggn2.10051)
Supplement: Supplementary file 1 — Appendix S1: Supplementary Note Supplementary Figure S1.1 Zana's descendants [file GGN2-2-e10051-s001.pdf]

Electronic supplementary material

The genomic origin of Zana of Abkhazia

Zana's story according to the witness accounts, written sources and earlier studies

Introduction

Written accounts about human-like creatures inhabiting forests in the Caucasus are traced in time as far back as to the10th century AD (1). Almost every ethnicity in the region has its own stories about such encounters. Traditions have it that the creatures inhabited the region prior to its population by ancestors, but were decimated in conquest for the living space and driven out to desolate areas (2). Yet, numerous accounts have been recorded about more recent encounters, occasional killings of the creatures by hunters, or their catching and taming with subsequent living in captivity in noblemen's households (1–4). While names given to the creatures vary with languages spoken in the region, they are invariably translated as a man of the woods, a wildman, or a hairy man (*meshe-adam* in Azerbaijani, *tskhiss katsi* in Georgian, *agatch-kishi* in Karachai, *almasty* in Kabardino-Balkaria, etc.). Cryptozoology enthusiasts consider such creatures relic hominids. In Abkhazia, the Western Caucasus, relict hominids are called *ochokochi* (in Mingrelian) or *abnauayu* (in Abkhazian).

One of such stories about Zana acquired worldwide recognition due to well documented attention of many academic and amateur researchers. According to such stories, Zana was a female *abnauayu* who was caught, tamed, and was living among humans until her death in the 1880s or 1890s. She was buried near the village of Tkhina (approximate coordinates 42.8821°, 41.5649°), on the Mokvi river, in the Ochamchire district of Abkhazia, about 78 kilometers from its capital Sukhumi. Local centenarian witnesses told the story to zoologist Alexander Mashkovtsev in 1962, who investigated the case in more detail. At the time, verbal accounts about Zana could be found in almost every household in the village and her life and death was still within memory of many 80+ year old elders. Prominent historian and sociologist Boris Porshnev, who joined the investigation in 1964, reported the story and summarized the collected evidence in his books (2,4).

Zana's story according to witness accounts and local stories

Details of her capture, such as the date and location, remain vague. According to one account she was captured in Zaadan forest, other accounts mention the Ochamchire coast or even the Adjara coast in Georgia bordering Turkey. The Adjara version better conforms with possible meaning of her nickname Zana, which could be derived from Georgian (ზანგი, zangi) broadly meaning "black-skinned person".

Hunters purportedly tracked her down and used age-old techniques to lure in a trap and counter her furious fighting. She was subdued only after several strong male hunters repeatedly pounded her with cudgels, gagged her mouth with felt, and shackled her legs to a log. After being sold multiple times as a slave she was presented to a nobleman Edgi Genaba, who took her, still shackled and chained, to his estate in the village of Tkhina. For the first three years she was lodged in a strong enclosure with log walls, for she acted like a wild beast. Nobody dared to venture in and food was thrown to her. She dug herself a hole in the ground and slept in it. She gradually became calmer and after three years she was moved to a wattle-fence enclosure under an awning away from the house. Villagers teased her with sticks thrust through the wattle-fence, and she would snatch them with fury, bare her teeth and howl. Being tethered at first, she was later let loose to wander about, yet she never went far from the place where she was accustomed to receiving her food. She could not tolerate warm rooms and stayed outdoors all year around, sleeping in a hole that she made herself under the awning. In the summertime she liked to lie in a cool pool side by side with buffalos. She swam in the cold mountain river and preferred to walk naked even in winter. She tore the dresses given to her but showed more tolerance toward a loincloth.

Over decades that she lived with people she could not speak. Zana did not learn a single Abkhaz word: she only made inarticulate sounds and mutterings, and cries when irritated. Sometimes, she would give a spontaneous laugh, baring her big white teeth. The latter were so strong that she easily cracked the hardest walnuts. At night she used to roam the surrounding hills. She wielded big sticks against dogs and on other perilous occasions. Zana had a curious obsession for playing with stones, knocking one against another and splitting them. Women were afraid of her and came near only when she was in a peaceful mood; when angry she presented a scary sight and could even bite, but in general did not show unprovoked aggressiveness toward villagers. Adults used her as a bogy figure with children, although Zana never actually attacked children.

Zana recognized her name and could perform mainly simple domestic tasks, like striking fire from flint onto lichen tinder and fanning it, turning hand millstones, bringing home firewood and water, carrying sacks to and from the watermill, or pulling her master's high boots off.

Her skin was black, or dark grey, and her whole body covered with reddish-black hair, with the exception of her face, feet and palms. The hair on her head was tousled and thick, hanging mane-like down her back. Zana was about 2 meters tall, massive and broad, with huge breasts and buttocks, muscular arms and legs, and fingers that were longer and thicker than ordinary human fingers. She could splay her toes widely and move apart the big toes. She had a flattened nose with turned out large nostrils and a snout-like prognathic lower part of the face with high cheekbones, muzzle-like jaws, a wide mouth with large white teeth. A low forehead with the hairline beginning from shaggy thick brows, an unusually high occiput, and eyes with a reddish tinge. But the most terrifying was her facial expression, which was purely animal, not human. No one could recall her smiling or crying. She lived for many years without showing signs of ageing: no grey hair, no falling teeth, keeping strong and fit as ever.

Her strength was enormous. She could outrun a horse, and swim across the wild Mokvi river even when it violently rose in high tide. Seemingly without effort she lifted with one hand an 80 kg sack of flour and carried it uphill from the water-mill to the village. Although clumsy like a bear, yet she easily climbed trees to get fruits; to gorge herself with grapes she would pull down a whole vine growing around the tree. She ate whatever was offered to her, including hominy and meat, with bare hands and enormous

gluttony; she was allowed to have wine, which brought her into a good mood and then she slept in a swoon-like state.

African hypothesis of Zana's origin

The accounts of her black or dark skin prompted the researchers to examine the possibility that Zana was simply of sub-Saharan African descent, who had been misidentified as *abnauayu* because of her unusual appearance in the eyes of the villagers. Today, Abkhazians of African descent or "Afro-Abkhazians", also known as African Caucasians, constitute a minor ethnicity living in compact settlements on the Caucasian coast of the Black Sea, mainly in Adzyubzha, a rural settlement in the Ochamchire district of Abkhazia, and in the surrounding villages (5). Their historic origins are still a matter of dispute. Under one hypothesis, a few hundred slaves were bought in the 17th century by a Georgian ruling family of the Principality of Abkhazia, and brought into the region to work on citrus plantations. Under another account, the Afro-Abkhazians came from Ethiopia by an unknown route. One legend refers to the Russian Tzar Peter the Great, who imported many Africans to Russia. Those unable to acclimate to harsh St. Petersburg weather were subsequently sent as gifts to Abkhasian princes, and thus were founders of the modern Afro-Abkhazian population. The witnesses were questioned whether Zana could be one of such slaves or their descendants who run away and became feral living in forest wilderness. All witnesses questioned unanimously rejected such possibility, confirming that they have seen local people of sub-Saharan African descent (presumably Afro-Abkhazians), and Zana was nothing similar. Rather, in their eyes she was not human, but a hairy beast that remained in the animal state despite all attempts to civilize her.

Zana's family

During her time in the village Zana became pregnant several times from local men. According to the local stories, Zana gave birth without assistance and, washed her newborns in the ice-cold water of a local spring (that still carries her name). Initially the infants did not survive such experience. Thus, after the villagers noticed that they began to take infants away from her in a good time, and reared them in their families. The adopted children grew up to become active members of the local society. All married in time and had their own children.

Zana's eldest son, Dzhonda, had about 9 daughters, and died between 1930 and 1940, and was buried in the village of Pokveshi in Abkhazia.

The eldest daughter of Zana was Gamasa who had a son (Goog) and a daughter (Chaka). She died in 1925 at the age of approximately 60 years, and was buried in the village of Tkhina in the Genaba family cemetery in Tkhina.

The second daughter Kodzhanar also had two daughters (Kuas and Maka), the date and place of her death remains unknown. Kuas was visited in the village of Mokua or Merkula in 2010 by a journalist, who obtained from her a photograph of her mother Kodzhanar (Figure S1.1).

The youngest son Khwit married twice and had one daughter (Tanya) from the first marriage and daughter Raya and son Shaliko from the second marriage (Figure S1.1). He died in 1954 at the age of 65-70 years and was buried in the Genaba family cemetery (rumored next to his mother, Zana); his burial

site was still clearly marked at the time of the studies. Rumor had it that both Gamasa and Khwit were sired by Edgi Genaba, which explains their final resting places in the Genaba family cemetery.
Many witnesses in Tkhina remembered and could describe very well both Gamasa and Khwit. Both were very strongly built, but otherwise inherited rather little of mother's facial appearance. According to the locals, Gamasa was about twice as stronger than an average male, had a very dark skin and noticeable hypertrichosis. Khwit also had a darker than usual skin, and larger lips than usual for the local population, but his hair was straight and stiff, as opposed to the curls characteristic of people of sub-Saharan African ancestry. Otherwise he was described by his fellow villagers as little different from the average villager. He was extremely strong, difficult to deal with and quick to pick a fight. In one of such fights with his fellow villagers he lost his right hand, yet his left hand sufficed him to mow and do other work on a collective farm, and even climb trees.

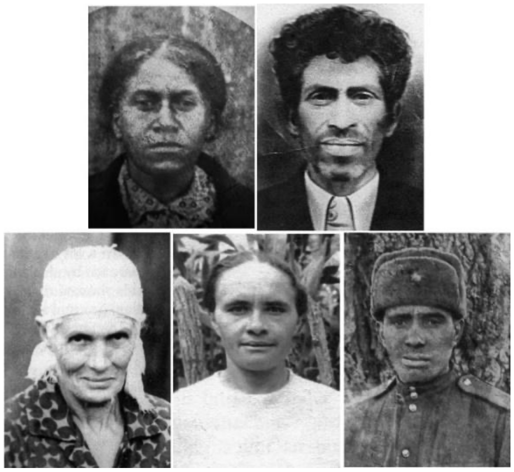

Supplementary Figure S1.1 Zana's descendants. Top panel – Zana's children, from left to right: Kodzhanar, Khwit. Bottom panel: Khwit's children, from left to right: Tanya, Raya, Shaliko.

Excavations

During 1964-65 studies, with permission from authorities and Zana's living descendants, Boris Porshnev made several attempts to find Zana's burial site in the Genaba's family cemetery. Excavation targets were identified with the assistance of centenarian witnesses and Kenton Genaba, the last member of the Genaba family, who was 79 years old at the time. However, these attempts were not successful. After face reconstruction made by M. Gerasimov, the famous founder of the forensic sculpture methodology, one of the excavated skulls was identified as likely one of Zana's deceased grandchildren

because of noticeable similarity of the reconstructed face with faces of two living Zana's grandchildren whom Boris Porshnev met earlier (2,4).

Subsequent studies in 1971-2015 expanded the tree of Zana's descendants with continuing attempts to find Zana's burial site. Photographs of some known Zana's descendants are shown on Figure S1.1. Tanya, Khwit's daughter from the first marriage, was visited in 1971 and provided a photograph of her father Khwit. Raya, Khwit's daughter from his second marriage, was also visited in 1971 and provided a photograph of her brother Shaliko (then deceased). Kodzhanar's photograph was brought by a journalist who in 2010 visited Kuas, one of Kodzhanar's daughters, in the village of Mokua (or Merkula) in the Ochamchire district of Abkhazia.

Although witnesses advised that Khwit was buried somewhere near his mother's burial in the Genaba's family cemetery, excavations near his burial completed in 1971 did not yield the desired result as excavated remains did not appear morphologically as consistent with Zana's appearance described by witnesses. Therefore, it was decided to exhume Khwit's remains, since his burial site was clearly marked. The exhumed skull (designated Tkhina-71) exhibited impressive anatomical peculiarities, in particular massive zygomatic bones, mandible and deep temporal fossa implying very massive temporalis and masseter muscles and strong jaws (Figure S1.2).

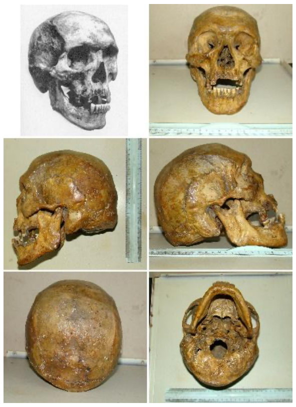

Supplementary Figure S1.2 Tkhina-71 skull.

The second, more extensive and systematic excavation was attempted in 1975 during joint expedition of the Institute of Ethnology and the Institute of Archaeology under auspices of USSR Academy of Sciences, and the magazine "*Around the World*". The area around Khwit's burial site was divided on squares of 2 x 2 meters and a total seven burial sites were excavated. One skeleton attracted attention because of its unusual burial position which was inconsistent with the traditional burial practices (Figure S1.3).

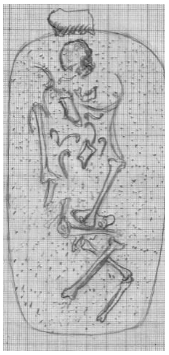

Supplementary Figure S1.3 Sketch of the untraditional burial where Tkhina-75 skull was found.

The body was not buried in a coffin and it was laid on its left side with bent legs in a curled-up position. The burial was old as bones suffered significant damage from ground water and the skull (designated Tkhina-75) collapsed due to decomposition of the parietal and temporal bones on its right side. A highly unusual feature of the skull was prominent prognathism of its lower part. A small mirror found near the skull indicated a female burial.

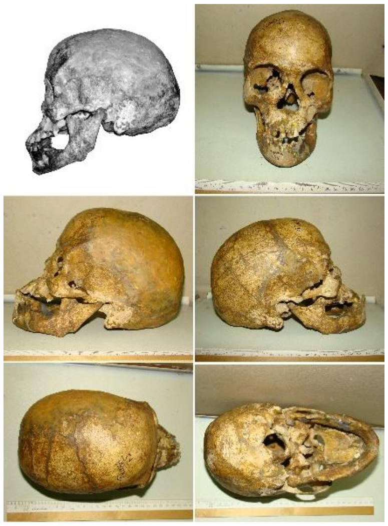

Supplementary Figure S1.4 Tkhina-75 skull.

Anthropological analyses

Both the Tkhina-71 and Tkhina-75 skulls were transferred to Moscow, Russia, for further studies. The skeleton remains from both burials were stored in Sukhumi, the capital of Abkhazia, in the Museum of Natural History, and perished when the museum was burned to the ground in the ethnic conflict that engulfed Abkhazia in 1992.

Comparative craniometric studies of the Tkhina-71 skull and skulls of modern Abkhaz males from the collections of the Moscow State University Institute of Anthropology were conducted by anthropologists M. Kolodieva and M. Gerasimova (6) and reported the following conclusions (translated by M. Heaney, 1988):

1. The Tkhina-71 skull is characterized by a significant increase in absolute measurements with some small deviations in proportions in comparison with the mean Abkhaz type.

2. The facial section of the skull is significantly larger in comparison with the mean Abkhaz type. It is characterized by a sharp horizontal profile in the sub-nasal region with strong development of the canine fossa and orthognathism.
3. The Tkhina skull exhibits an original combination of modern and some "archaic" features. The specificity of the deviation of the Tkhina skull (from the mean in the Abkhaz series) in comparison with the deviation of the most massive skull (No. 3896) in the series was revealed, in respect of the absolute size of the measurements and their proportions, and also in respect of the composition of the features themselves. The Tkhina skull is characterized by brachycephaly, hypocephaly, the low height of frontal curvature, the great length of the facial section and the marked horizontal profile (in comparison with the large skulls of the Abkhaz series). The long axis of the skull was significantly larger, as were the malar axis, the breadth of the orbit, the projection of the glabella and the brow ridges.
4. All the measurements and indices of the superciliary cranial contour are greater not only than those of the mean Abkhaz series, but also than those of the maximum size of some fossil skulls studied (or rather were comparable with the latter).
5. The Tkhina skull approaches closest the Neolithic Vovnigi II skulls of the fossil series in a number of the features of the brain case and facial region, and also in the development of the superciliary contour.

Restoration of the Tkhina-75 skull was conducted at the Laboratory of Plastic Reconstruction, the Institute of Ethnography, USSR Academy of Sciences (now the Institute of Anthropology and Ethnology of Russian Academy of Sciences) under supervision of G. Lebedinskaya. The restored Tkhina-75 skull (Figure S1.4) was examined in 1976 by A. Zubov, who summarized his findings as follows (translated from Russian by Vladimir Yamshchikov).

The skull was of a human female of an average size. By the appearance of the cranial sutures, it can be said that the woman died at the age of about forty years, although based on the teeth wear, she could be at the age of fifty. Some features are peculiar: a very strong forward protrusion (alveolar prognathism) of the upper jaw, a shallow sloping palate, a strongly protruding occiput, a wide nasal opening with fossae prenasalis. The teeth are very uneven. In the area of the upper canines, there is a substantial distension of the jaw bones, which may indicate large canines (they are markedly worn). Due to this, a number of upper jaw teeth are almost Π-shaped. The lower jaw is somewhat too heavy for such a face, its bone is thicker, especially in its anterior part (symphysis). Noteworthy is the strong prognathism and slanted planting of the teeth with forward protrusion, which, apparently, resulted in their specific wear pattern.

Some of these features could be considered archaic if they were not combined with such progressive features as absence of the supraorbital ridge, large mastoid processes, which although indicative of strong musculature are characteristic specifically for a modern human, the normal for a modern human structure of teeth, small thickness of bones.

In general, the skull is quite peculiar: according to many of these features, it can be generally attributed to a sub-Saharan African type, but this is partly contradicted by the large orbital height and overall large facial height.

Overall, the skull is interesting particularly if one bears in mind the location of its discovery. However, the African features are not that surprising in connection with the existence of a small African population in Abkhazia. The corresponding admixture is traceable in some places in the modern population there.

From the anthropological point of view, it makes sense to continue searching for such material, even though interpretation of the skull is still premature.

Facial reconstruction on the Tkhina-75 skull using M. Gerasimov's methodology of forensic sculpture was done in 1976 by G. Lebedinskaya and yielded a portrait of a middle-age woman of likely African ancestry, which in general agreed with conclusions from anthropological studies. The portrait was adapted in 2013 by an amateur artist Roman Moskalik taking into consideration Zana's appearance according to witness accounts (Figure S1.5).

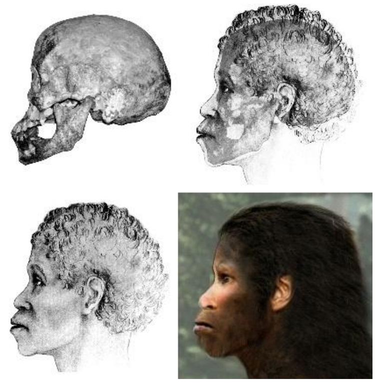

Supplementary Figure S1.5 Face reconstruction from Tkhina-75 skull (top left) by G. Lebedinskaya. Bottom right – artistic reconstruction after consideration of witness accounts.

Comparison of Tkhina-71 and Tkhina-75 skulls (Figure S1.6) revealed a number of morphological similarities, such as large zygomatic bones which suggested at least some potential relatedness. However, the conclusion that Tkhina-75 skull belonged to a female of sub-Saharan African origin did not support the hypothesis that she was Khwit's mother Zana. Some anthropologists who examined Khwit's photograph (Figure S1.1) consistently defined him as belonging to the "Aboriginal Australian" type. Therefore, search for Zana's burial continued. The last attempt of finding Zana's burial was undertaken in 1978 but did not yield any additional finds.

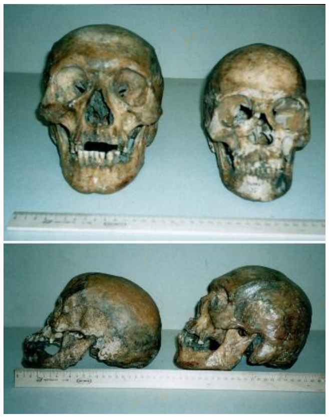

Supplementary Figure S1.6 Tkhina-71 (upper-left and lower-right) and Tkhina-75 (upper-right and lower-left) skulls.

Previous DNA analyses

To obtain a more definitive answer about the relatedness of Tkhina-71 and Tkhina-75 skulls bone samples from both skulls were taken to a laboratory in the New York University headed by geneticist T. Disotell. The study showed in 2006 that a fragment of human mtDNA was amplified by PCR from both bone samples. While experimental details of that study are not available, it was determined that haplotypes of detected mtDNA were likely the same and did not differ from haplotypes present in the region. This supported the hypothesis that Tkhina-71 and Tkhina-75 skulls belonged to maternal relatives, however, the results were likely erroneous due to contamination of aDNA in bone samples.

To overcome the potential issues of contamination, DNA was extracted from theeth samples of Tkhina-71 and Tkhina-75 and sequenced in 2012 using Illumina's NGS methodology by Vladimir Yamshchikov. Full mtDNAs of both individuals were reconstructed using reference assembly against

human rCRS mtDNA (NCBI acc. No. NC_012920) and were found to be identical belonging to the cluster of haplogroup L2b. Therefore, it was concluded that Tkhina-71 (Khwit's) and Tkhina-75 (the prognathic female's) skulls belonged to maternally related individuals.

Another attempt to employ genetic methods was undertaken in 2013, when Oxford geneticist and writer Bryan Sykes, with the support of Discovery channel (Discovery, Inc.), analyzed DNA isolated from teeth of both skulls and compared with DNA isolated from several Zana's descendants (neither of whom was of Zana's direct maternal descendance) and unrelated people living in Abkhazia (7). The book reports that genetic analysis of DNA extracted from Khwit's (Tkhina-71 skull's) tooth revealed that Khwit's mtDNA belonged to the haplogroup L2c. Nuclear genetic analysis also led to a number of interesting conclusions. Contrary to interpretations by the mainstream media that Zana was simply an escaped African slave, the author concludes that she was no slave from Africa but an individual with genetics that tells much more about the population from which she originated (7), p. 306: "Zana's ancestors could have left Africa before the Laran exodus of 100,000 year ago…they might well be still there [in Caucasus mountains] to this day, living as they have for millennia somewhere in the wild valleys that radiate from the eternal snows of Elbrus…"

Unfortunately, the book provides no experimental details and DNA sequence data from the performed genetic studies, which makes uncertain the extent of sequencing characterization of Khwit's mtDNA and the factual basis of haplogroup identification. In addition, the book does not report any results on genetic characterization of DNA extracted from the Tkhina-75 skull; in personal communication B. Sykes mentioned that sequencing analysis revealed a mtDNA haplotype clearly different from Khwit's haplotype, probably representing one of haplotypes present in the area. In any case, if not resulting from contamination with modern DNA, this indicated lack of relatedness between Tkhina-71 and Tkhina-75 skulls.

Material and Methods

Data generation

Initially, 100 mg dentine was sub-sampled from a tooth from both individuals, into two different DNA LoBind tubes (Eppendorf, Hamburg, Germany) and DNA was extracted using a MinElute (Qiagen, Hilden, Germany) spin column following the protocol of Loog et al. 2019 (8). Two double-stranded BGISeq libraries were generated from 32 µl of each extract following the BEST protocol, using adapters compatible with BGI sequencing according to Mak et al. 2017 (9). Each library was then double indexed and amplified using PfuTurbo Cx Hotstart (Agilent Technologies, Santa Clara, CA, United States) and purified using QIAquick PCR Purification Kit (Qiagen), following manufacturer's guidelines. The amplified libraries were quantified using the High-Sensitivity DNA Assay on an Agilent 2200 TapeStation (Agilent Technologies, Palo Alto, CA, USA). The amplified libraries were sequenced on 1 lane of a BGISEQ-500 platform with PE100 chemistry.

Due to poor DNA preservation of the teeth, petrous bones were subsequently sampled, from both Khwit and putative Zana individuals (henceforth referred to as 'Zana' when referring to the hypothetical identification, and Zana when referring to the concrete individual). They were sampled by isolating the densest part of the cochlea, which contains the highest endogenous DNA fraction (10,11). The drilled bone samples were divided into two different DNA LoBind tubes (Eppendorf) per individual, each containing 100-200mg of bone. DNA extractions were performed following a modified silica-in-solution protocol (12–14) and eluted in 64 µl of Qiagen's EB buffer. Two double-stranded BGISeq libraries were generated from 32 µl of each extract following the BEST protocol, using adapters compatible with BGI sequencing according to Mak et al. 2017 (9). Each library was then double indexed and amplified using

Amplitaq Gold (Thermo Fisher Scientific) and purified using SPRI beads as described elsewhere(15). The amplified libraries were quantified using the High-Sensitivity DNA Assay on an Agilent 2200 TapeStation (Agilent Technologies, Palo Alto, CA, USA). The amplified libraries were sequenced on 1 lane of a BGISEQ-500 platform with SE100 chemistry.

Bioinformatics analysis and quality assessment

We used the PALEOMIX pipeline (16) to trim and map the sequencing reads. In brief, BGI adaptor sequences and stretches of Ns at both ends of the reads were trimmed from all of sequences using AdapterRemoval v2.2 (17), keeping only reads with a minimum length of 30 bases. Subsequently the trimmed sequences were mapped against the human reference genome build GRCh37 and the revised Cambridge reference sequence (rCRS, NCBI accession number NC_012920.1) using the BWA v0.7.15 with "aln" algorithm (18) with the seed disabled allowing higher sensitivity (19). The aligned sequences were filtered for mapping quality 30 and sorted using Samtools (20). Duplicate sequences were removed by Picard MarkDuplicates (http://picard.sourceforge.net).

We used contamMix for estimating the authentic fraction of DNA in ancient samples. The method relies on the reconstructed mtDNA consensus sequences of ancient samples. We aligned DNA reads from both individuals to the human mitochondrial reference genome: revised Cambridge Reference Genome (rCRS) with the same parameters as for the whole genome mapping. Base quality ≥20 and mapping quality ≥30 were applied and only SNPs and sites with at least 10X coverage were considered. The details of contamMix are described elsewhere (21). In addition, for the male individual we also used the X-chromosome based contamination method (22) implemented in ANGSD (23) by applied mapping quality ≥30 and base quality ≥20 filters. The reported values are based on the maximum likelihood values using the unbiased sampling-based approach, i.e. "Method1" in ANGSD.

Kinship analyses

We assessed the relationship between the two samples in several ways. Firstly, to account for the hypothesis that Khwit's mother was not genetically local to the region, thus Khwit's genome would be a mosaic of his local father, and divergent mother, we used PC-relate as implemented in PCAngsd (24,25) so as to account for the distinct parental lineages.

Secondly, a Beagle (26) genotype likelihood file was created by merging both individuals' beagle files and the Affymetrix Human Origins SNP array panel of worldwide populations. The principal components were calculated using PCAngsd inputting the new merged beagle file and selecting the -kinship flag. This generated a kinship matrix which was then analyzed in R for depicting relatedness based on the classification criteria proposed by Manichaikul et al. (27), where a pair of individuals is classified to have a first degree relationship if their estimated kinship coefficient is in the interval (0.177-0.354).

Thirdly, as the kinship estimator from PC-relate cannot distinguish between parent-offspring and full siblings, an additional statistic proposed by Lee (28), R0, was used to differentiate between them: the expected value for R0 for parent-offspring pairs is 0, while for full-siblings the value is above 0 (adhoc cut-off of 0.02) (29). The statistic R0 was calculated by first generating a Site Allele Frequency Likelihood file (saf) in ANGSD (http://www.popgen.dk/software/index.php/IBSrelate) for each individual based on only transversion biallelic sites of the 1000 Genomes project. For this analysis we

used only sites with a depth of 5 for each individual (-minIndDepth 5 filter in ANGSD). Then an IBS sharing matrix was obtained by estimating the two-dimensional Site Frequency Spectrum (2d-SFS) from real-SFS implemented in ANGSD and R0 was inferred from the results.

Population genetics

Next, we assessed the genetic relationship between 'Zana', Khwit and other populations, by merging the shotgun sequencing data from the historical individuals with the Affymetrix Human Origins SNP array panel of worldwide populations (30,31). We extracted autosomal genotypes at a subset of 593,102 SNPs that were also included in the "1240K" capture panel (32). To test the "non-human" cryptozoological hypothesis of Zana's genetic ancestry, we also included data from three archaic humans (two Neanderthals (33) and the Denisovan (34)) and chimpanzee genotypes. In addition, given the geographic proximity, we also merged ancient human genome data from two Caucasus hunter-gatherers (CHG) (SATP-13,300 years old and KK1-9,700 years old) originally excavated in the South Caucasus (35) for comparison.

To obtain the genotypes of the ancient samples, we used the "bcftools mpileup" command followed by a single read sampling of the majority allele for each of the sites present in the relevant reference dataset, with mapping and base quality ≥30.

Based on the pseudohaploid individuals from the "Human Origins" merge we conducted unsupervised maximum likelihood-based clustering analysis with ADMIXTURE (36) by pruning the dataset for linkage disequilibrium using plink v1.9 (37) with the settings (--indep-pairwise 50 10 0.1). We used two panels for each of the historical individuals: (i) subset of worldwide populations with 300 individuals and 190,111 markers after using genotyping rate (--geno) and minor allele frequency > 1% (--maf 0.01) filters in plink v1.9. (ii) African populations from the HO panel with 232 individuals (randomly chosen ca. five individuals from each group) and 270,311 markers after applying the same filters as for the previous subset. The program pong (38) was used to identify and visualise the best run for each K and similar components between different Ks. We also conducted clustering ADMIXTURE analysis in "supervised" mode based on 300 replicates with different seeds using 13 African populations to estimate 'Zana's' ancestry components.

We performed principal components analysis (PCA) using plink v1.9 with the ancient genomes projected onto the modern variation. We used the first thirty eigenvectors of PCA as input for the uniform manifold approximation and projection (UMAP) analysis using the "umap" R package.

D-statistics estimates were calculated using the ADMIXTOOLS (39) and R package "admixr"(40). Only 175,148 transversion sites were considered for this analysis.

The maximum likelihood phylogenetic tree of 'Zana' and the African populations were constructed with TreeMix (41). We only considered 101,799 transversions sites with the Altai Neanderthal as the outgroup and used 20 African populations for readability purposes. We randomly chose five individuals from each group. The presented tree is the consensus of one hundred replicates with random seed values. We used 100 SNPs per block (-k parameter) which resulted in 1017 blocks.

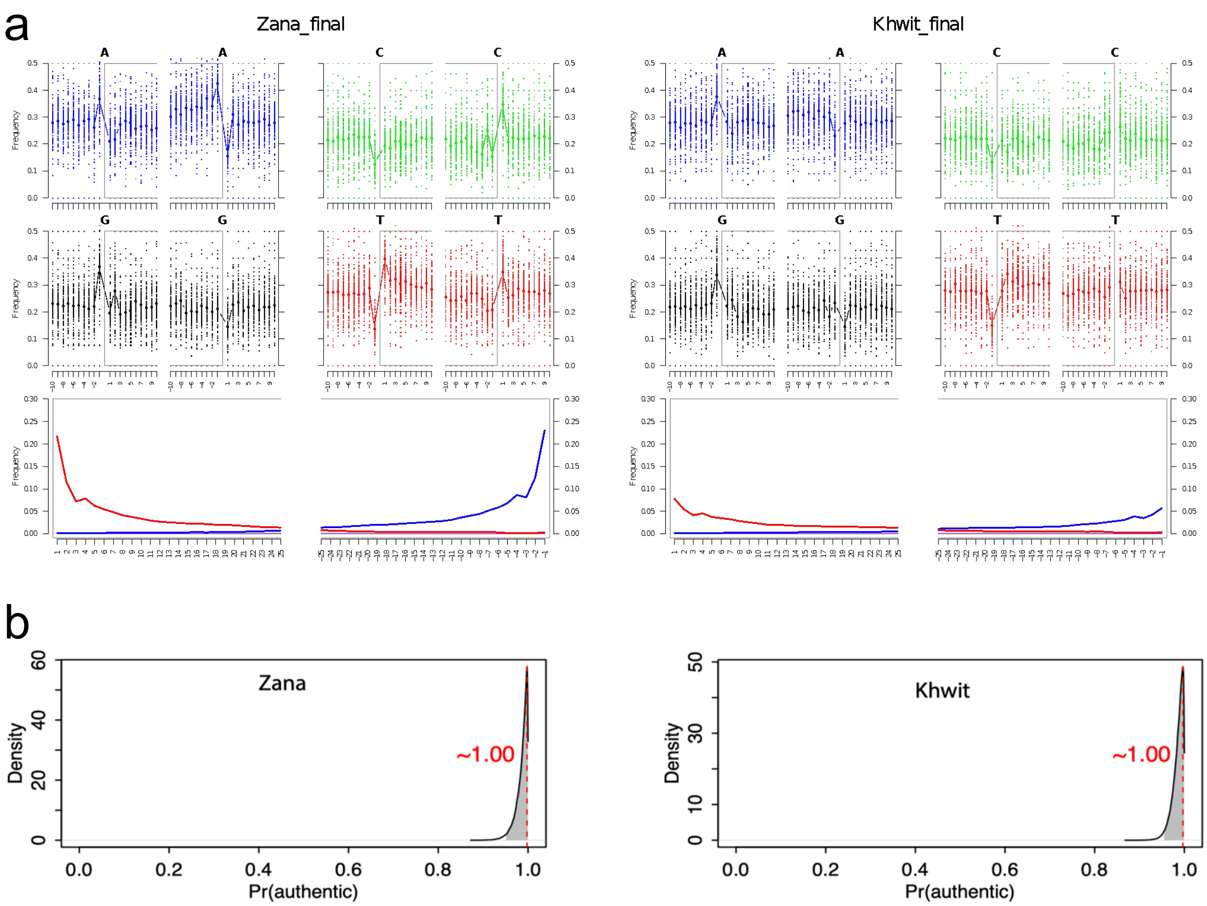

Supplementary Figure S2 Quality control of ancient/historical DNA libraries

a) Typical ancient DNA damage patterns of both individuals. **b**) Probability of authentic human DNA based on mitochondrial contamination estimates by contamMix.

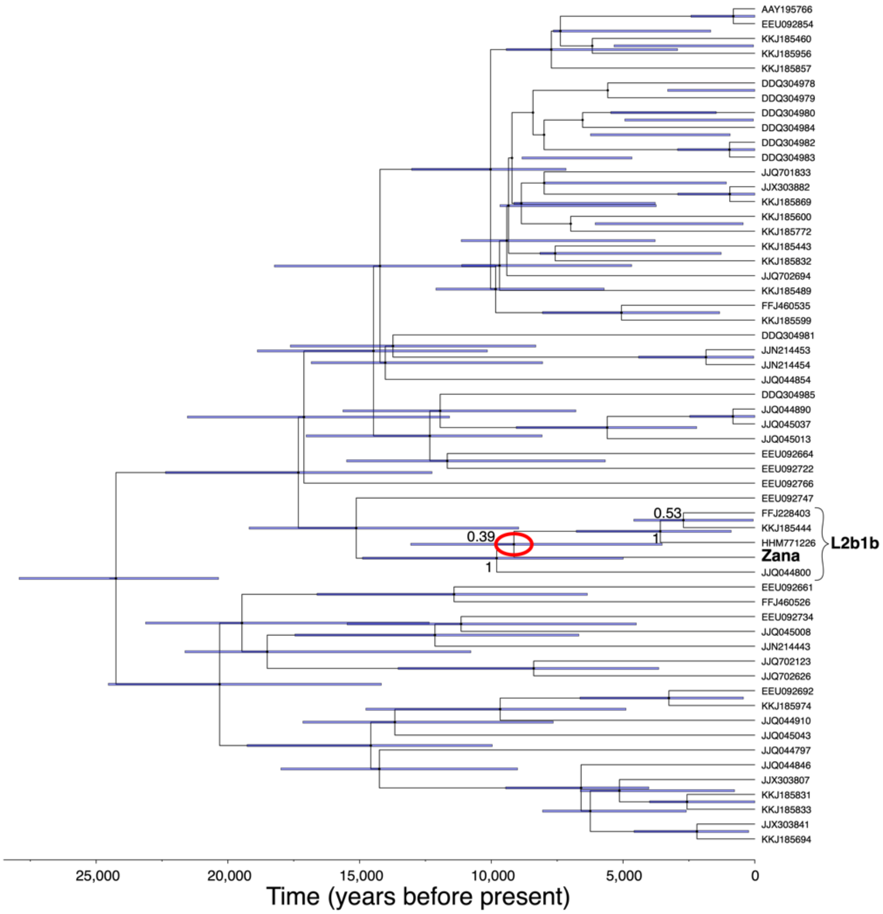

Supplementary Figure S3 Phylogenetic tree of L2b haplogroup lineages

The tree is based on 57 present day individuals (42). The divergence time of Zana's maternal lineage was estimated to be ca. 9,800 ya (3,515 - 13,000; 95% highest posterior density intervals), though the support values for that node was only 0.39. However, all five L2b1b lineages coalesce ca. 10,000 year ago (ca. 5,000 - 15,000, 95% highest posterior density intervals) with a 100% bootstrap support estimates, indicating that Zana's maternal lineage is not older than 10-15 ky.

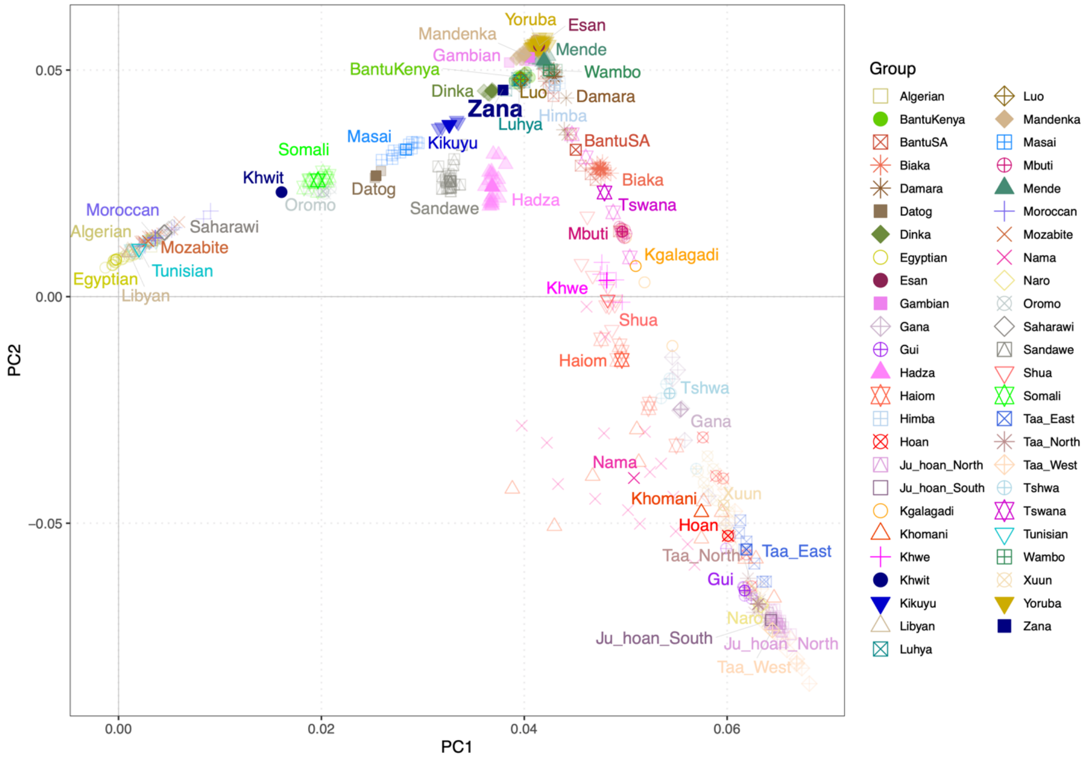

Supplementary Figure S4 PCA with African groups

Zana and Khwit are analyzed along with African populations from the Human Origins panel. Khwit's intermediate location on the PCA plot was expected (as in Figure 2 of the main text), due to his 50% African (from Zana) and 50% European/South-Caucasian genetic ancestries.

References

1. Bayanov D. In the Footsteps of the Russian Snowman: A Record of Investigation. Moscow, Russia: Crypto-Logos Publishers.; 1996.
2. Porshnev B. Sruggle for Troglodytes (In Russian). Prostor; 1968.
3. Burtsev ID. Hominology in Action. Book One: In Search of Evidence. Yeti, Snowman, Almasty, Bigfoot & Co: Russia and Beyond. Moscow: Crypto-Logos Publishers.; 2016.
4. Heuvelmans B, Porshnev B. L'Homme de Néanderthal est toujours vivant. Paris: Plon.; 1974.
5. Abkhazians of African descent [Internet]. Wikipedia. [cited 2020 Nov 7]. Available from: https://en.wikipedia.org/w/index.php?title=Abkhazians_of_African_descent&oldid=987482720
6. Burtsev, ID and Kolodieva, MA. Results of preliminary investigation of a skull from the village of Tkhina, Abkhazia. Moscow: Nauka.; 1987.
7. Sykes B. The Nature of the Beast: The first genetic evidence on the survival of apemen, yeti, bigfoot and other mysterious creatures into modern times. London: Hodder & Stoughton; 2014.
8. Loog L, Thalmann O, Sinding M-HS, Schuenemann VJ, Perri A, Germonpré M, et al. Ancient DNA suggests modern wolves trace their origin to a Late Pleistocene expansion from Beringia. Mol Ecol. 2020 May;29(9):1596–610.
9. Mak SST, Gopalakrishnan S, Carøe C, Geng C, Liu S, Sinding M-HS, et al. Comparative performance of the BGISEQ-500 vs Illumina HiSeq2500 sequencing platforms for palaeogenomic sequencing. Gigascience. 2017 Aug 1;6(8):1–13.
10. Pinhasi R, Fernandes D, Sirak K, Novak M, Connell S, Alpaslan-Roodenberg S, et al. Optimal Ancient DNA Yields from the Inner Ear Part of the Human Petrous Bone. PLoS One. 2015 Jun 18;10(6):e0129102.
11. Gamba C, Jones ER, Teasdale MD, McLaughlin RL, Gonzalez-Fortes G, Mattiangeli V, et al. Genome flux and stasis in a five millennium transect of European prehistory. Nat Commun. 2014 Oct 21;5:5257.
12. Gilbert MTP, Bandelt H-J, Hofreiter M, Barnes I. Assessing ancient DNA studies. Trends Ecol Evol. 2005 Oct;20(10):541–4.
13. Damgaard PB, Margaryan A, Schroeder H, Orlando L, Willerslev E, Allentoft ME. Improving access to endogenous DNA in ancient bones and teeth. Sci Rep. 2015 Jun 17;5:11184.
14. Allentoft ME, Sikora M, Sjögren K-G, Rasmussen S, Rasmussen M, Stenderup J, et al. Population genomics of Bronze Age Eurasia [Internet]. Vol. 522, Nature. 2015. p. 167–72. Available from: http://dx.doi.org/10.1038/nature14507
15. Rohland N, Reich D. Cost-effective, high-throughput DNA sequencing libraries for multiplexed target capture. Genome Res. 2012 May;22(5):939–46.
16. Schubert M, Ermini L, Der Sarkissian C, Jónsson H, Ginolhac A, Schaefer R, et al. Characterization of ancient and modern genomes by SNP detection and phylogenomic and metagenomic analysis using PALEOMIX. Nat Protoc. 2014;9(5):1056–82.
17. Schubert M, Lindgreen S, Orlando L. AdapterRemoval v2: rapid adapter trimming, identification, and read merging. BMC Res Notes. 2016;9:88.
18. Li H, Durbin R. Fast and accurate short read alignment with Burrows–Wheeler transform. Bioinformatics. 2009 Jul 15;25(14):1754–60.
19. Schubert M, Ginolhac A, Lindgreen S, Thompson JF, Al-Rasheid KAS, Willerslev E, et al. Improving ancient DNA read mapping against modern reference genomes. BMC Genomics. 2012;13:178.
20. Li H, Handsaker B, Wysoker A, Fennell T, Ruan J, Homer N, et al. The Sequence Alignment/Map format and SAMtools. Bioinformatics. 2009 Aug 15;25(16):2078–9.
21. Fu Q, Mittnik A, Johnson PLF, Bos K, Lari M, Bollongino R, et al. A revised timescale for human evolution based on ancient mitochondrial genomes. Curr Biol. 2013 Apr 8;23(7):553–9.
22. Rasmussen M, Guo X, Wang Y, Lohmueller KE, Rasmussen S, Albrechtsen A, et al. An Aboriginal Australian genome reveals separate human dispersals into Asia. Science. 2011 Oct 7;334(6052):94–8.
23. Korneliussen TS, Albrechtsen A, Nielsen R. ANGSD: Analysis of Next Generation Sequencing Data. BMC

Bioinformatics. 2014 Nov 25;15:356.
24. Conomos MP, Reiner AP, Weir BS, Thornton TA. Model-free Estimation of Recent Genetic Relatedness. Am J Hum Genet. 2016 Jan 7;98(1):127–48.
25. Meisner J, Albrechtsen A. Inferring Population Structure and Admixture Proportions in Low-Depth NGS Data. Genetics. 2018 Oct;210(2):719–31.
26. Browning SR, Browning BL. Rapid and accurate haplotype phasing and missing-data inference for whole-genome association studies by use of localized haplotype clustering. Am J Hum Genet. 2007 Nov;81(5):1084–97.
27. Manichaikul A, Mychaleckyj JC, Rich SS, Daly K, Sale M, Chen W-M. Robust relationship inference in genome-wide association studies. Bioinformatics. 2010 Nov 15;26(22):2867–73.
28. Lee W-C. Testing the genetic relation between two individuals using a panel of frequency-unknown single nucleotide polymorphisms. Ann Hum Genet. 2003 Nov;67(Pt 6):618–9.
29. Waples RK, Albrechtsen A, Moltke I. Allele frequency-free inference of close familial relationships from genotypes or low-depth sequencing data. Mol Ecol. 2019 Jan;28(1):35–48.
30. Lazaridis I, Patterson N, Mittnik A, Renaud G, Mallick S, Kirsanow K, et al. Ancient human genomes suggest three ancestral populations for present-day Europeans. Nature. 2014 Sep 18;513(7518):409–13.
31. Lazaridis I, Nadel D, Rollefson G, Merrett DC, Rohland N, Mallick S, et al. Genomic insights into the origin of farming in the ancient Near East. Nature. 2016 Aug 25;536(7617):419–24.
32. Mathieson I, Lazaridis I, Rohland N, Mallick S, Patterson N, Roodenberg SA, et al. Genome-wide patterns of selection in 230 ancient Eurasians. Nature. 2015 Dec 24;528(7583):499–503.
33. Prüfer K, Racimo F, Patterson N, Jay F, Sankararaman S, Sawyer S, et al. The complete genome sequence of a Neanderthal from the Altai Mountains. Nature. 2014 Jan 2;505(7481):43–9.
34. Meyer M, Kircher M, Gansauge M-T, Li H, Racimo F, Mallick S, et al. A high-coverage genome sequence from an archaic Denisovan individual. Science. 2012 Oct 12;338(6104):222–6.
35. Jones ER, Gonzalez-Fortes G, Connell S, Siska V, Eriksson A, Martiniano R, et al. Upper Palaeolithic genomes reveal deep roots of modern Eurasians. Nat Commun. 2015 Nov 16;6:8912.
36. Alexander DH, Novembre J, Lange K. Fast model-based estimation of ancestry in unrelated individuals. Genome Res. 2009 Sep;19(9):1655–64.
37. Purcell S, Neale B, Todd-Brown K, Thomas L, Ferreira MAR, Bender D, et al. PLINK: a tool set for whole-genome association and population-based linkage analyses. Am J Hum Genet. 2007 Sep;81(3):559–75.
38. Behr AA, Liu KZ, Liu-Fang G, Nakka P, Ramachandran S. pong: fast analysis and visualization of latent clusters in population genetic data. Bioinformatics. 2016;32(18):2817–23.
39. Patterson N, Moorjani P, Luo Y, Mallick S, Rohland N, Zhan Y, et al. Ancient admixture in human history. Genetics. 2012 Nov;192(3):1065–93.
40. Petr M, Vernot B, Kelso J. admixr—R package for reproducible analyses using ADMIXTOOLS. Bioinformatics. 2019;35(17):3194–5.
41. Pickrell JK, Pritchard JK. Inference of population splits and mixtures from genome-wide allele frequency data. PLoS Genet. 2012 Nov 15;8(11):e1002967.
42. Silva M, Alshamali F, Silva P, Carrilho C, Mandlate F, Jesus Trovoada M, et al. 60,000 years of interactions between Central and Eastern Africa documented by major African mitochondrial haplogroup L2. Sci Rep. 2015 Jul 27;5:12526.
