# Supplementary material for: The genomic origin of Zana of Abkhazia
Source: Adv Genet (Hoboken). 2021 Jun 14;2(2):e10051. doi: 10.1002/ggn2.10051 (PMC9744565; doi:10.1002/ggn2.10051)
Supplement: Supplementary file 3 — Supplementary TPR File [file GGN2-2-e10051-s003.pdf]

ADVANCED GENETICS
Open Access

The genomic origin of Zana of Abkhazia

Ashot Margaryan*, Mikkel-Holger S. Sinding, Christian Carøe, Vladimir Yamshchikov, Igor Burtsev,

M. Thomas P. Gilbert*

*Corresponding

Review timeline:	Date Submitted:	26-Apr-2021	
	Editorial Decision:	13-May-2021	Accept in Principle
	Revision Received:	21-May-2021	
	Accepted:	21-May-2021	

Editor: Myles Axton

Initial Editorial Evaluation	26-Apr-2021

Summary

Petrous bone DNA extracted from two deceased individuals (1890 and 1952) from Abkhazia, Caucasus. Comparison with human ancestry SNP panel, chimpanzee and two Caucasus hunter-gatherer samples from 13.3kya and 9.7kya. The individuals are of L2b1b mitochondrial haplogroup, clustering with L2b within 93 L2 samples, and the son (kinship coefficient in 1st degree range) of R1b1a1b1b (European or Western Asian) Y haplogroup. Unable to resolve whether Z was admixed East and West African or East African.

Scope

Do the research, methods or topics fit within the aims of this, or another journal?

Technically OK from anthropological and ancient DNA point of view and accurate with respect to population genetics. There is minimal evidence for transmission of hypertrichosis, but good documented evidence for idiosyncratic skull morphology. Ethically, there is participation from the local region and the family of the historical individuals.

Conceptual advance

What is already known in this area and related fields?

Egil Skallagrimsson, Thomas Jefferson, Romanoff family, Julia Pastrana pedigree.

What gap in knowledge motivates this research?

Ref 35 is not peer reviewed and the “African” origin of Zana’s DNA has not been published (author Yamshikov, unpublished tooth DNA).

1st Peer Review	27-Apr-2021 to 12-May-2021

Reviewer #1
This is an unusual and delicate research subject, but the authors demonstrate that it can be approached from a scientific -as well as ethical- perspective with the right methodology. They use common tools on ancient DNA research to generate two low-coverage genomes -those of Zana's son and Zana's herself- and analyse them along with modern reference panels with an approach from population genetics. They use PCA and Admixture, among other tools, to obtain evidence that this unfortunate

person was in fact a woman of an East African origin who was probably taken to the Caucasus as a slave during the Ottoman rule. I appreciate the Ethical statement the authors have included at the end of the manuscript that makes emphasis in the dignity this person deserved and that at least could have now.

1.1 They hypothesize that Zana was suffering from hypertrichosis, something that seems reasonable. This condition has been recently linked to HTC2 gene in the X chromosome (locus 7.31); considering they have the complete genome of Zana, although at low coverage, I was wondering if it would be possible to check for any unusual mutation in this gene (I am not sure if the condition has been linked to a particular genetic variant so far, probably not). Even if it is not possible to determine this, maybe they could mention the possibility for future studies, once the potential mutations are better known, because the genome would be publicly available. This would also be important from a human disease point of view and for new, potential cases with this rare skin disease.

Reviewer #2

This is a historical/forensic case study of a storied individual mistakenly mythologized as a non-human. The remains have been studied with earlier aDNA techniques, with mistaken conclusions. Therefore, there is an imperative to correct the record. The techniques used here are appropriate, current and reliably executed. The analysis is clear and the conclusions seem sound. There is evidence of a (probably East) African ancestry. The MS is well written and there is suitable supplementary material (mainly historical information).

Reviewer #3

Review of Margaryan et al. "The genomic origin of Zana of Abkhazia"

This is a solid study that demonstrates the power of whole genome sequencing from human remains to shed light on their ancestry and their genealogical relationships to contemporary reference individuals. Although the questions addressed are not ground-breaking, the manuscript is nonetheless a tour-de-force demonstration of how genetic data can be used to resolve questions of this kind and definitely worth publishing.

The manuscript has improved substantially from a version that I commented on several months ago and I have no major comments or criticisms.

Minor comments

3m1 lines 154-158: This analysis seems unnecessary. The fact that the haplotype belongs to L2b, and appears like any other sub-haplotype of that haplogroup, is sufficient to reject any claim of Zana's mtDNA sequence being in any way "archaic".

3m2 line 184: define "HO"

3m3 line 185: define "CHG (KK1 and SATP)"

3m3 line 187: "markers" can be deleted.

3m4 line 214: What does "generic" mean in this context?

3m5 line 223: Why were only transversions used in the Treemix analyses? Was this also done for all other analyses? I can see no reason to restrict the analysis to transversions. I cannot see why cytosine deamination would bias the relationship of Zana to any of the modern reference populations.

3m6 lines 225-229: The D statistic test is not really needed. The observation that Zana is African puts the idea that she was "archaic" to bed. However, there is no harm in including the D stat analysis.

1st Editorial Decision	13-May-2021

Editorial decision: Accept in principle, subject to the revisions suggested by the reviewers and journal formatting requirements

Editor's understanding of the reviews
Reviewer #1 Recommends Minor Revision
Reviewer #2 Recommends Accept
Reviewer #3 recommends Accept

Author's Response to 1st Review	21-Apr-2021

Reviewer comments	Editor recommendation	Author reply	Changes to Manuscript
1.1 hypertrichosis, something that seems reasonable. This condition has been recently linked to HTC2 gene in the X chromosome (locus 7.31); considering they have the complete genome of Zana, although at low coverage, I was wondering if it would be possible to check for any unusual mutation in this gene	ED1 Discuss whether reports that Zana and one son (of four sibs) long abundant body hair is consistent with either autosomal dominant or X-linked hypertrichosis syndrome and what level of genomic coverage might enable identification of a monogenic variant accounting for this observation. The ethical framing of whether living and deceased individuals should have to account for their visible differences where there is a biological or genetic etiology, and who is entitled to query their differences is important to discuss. Individuals have made their living from rare genetic traits, and individuals have died from rare and common differences from the majority in their location. The common reason Zana was misunderstood has emerged from this work (her continental ancestry). The rare remains with her.	We only speculate about the hypertrichosis with an attempt to explain the Zana's unusual appearance (body hair, strange gaze, lack of speech allegedly lower than average mental capacity…). This hypothesis is difficult to assess since the disease can have various causes such as genetic (congenital) and acquired (manifested together by other diseases).<br><br>Importantly, the X-Linked congenital hypertrichosis syndrome that the reviewer-1 is mentioning is likely a large insertion on the X chromosome which cannot be reliably assessed by short ancient DNA fragments especially with low coverage data.	
3m1 lines 154-158: This analysis seems unnecessary. The fact that the haplotype belongs to L2b, and appears like any other sub-haplotype of that haplogroup, is sufficient to reject any claim of Zana's mtDNA sequence being in any way "archaic".<br>3m6 lines 225-229: The D statistic test is not really needed. The observation that Zana is African puts the idea that she was "archaic"	ED2 Explain the purpose of this BEAST TMRCA analysis on the limited set of L2b samples. Is this analysis redundant, given that you know Zana's lineage is L2b, or were you looking for something else? Secondly, as the reviewer offers, the D stat analysis can remain.	The L2b lineage is ca. 25–30 ky old. While this might already be enough to reject the presence of any archaic mtDNA lineage we used the BEAST analyses to give us a more accurate age for the split time (and upper bound) of Zana's maternal line from the rest of mtDNA lines.	

to bed. However, there is no harm in including the D stat analysis.			
3m5 line 223: Why were only transversions used in the Treemix analyses? Was this also done for all other analyses? I can see no reason to restrict the analysis to transversions. I cannot see why cytosine deamination would bias the relationship of Zana to any of the modern reference populations.	ED3 Add a sentence why the analysis was limited to transversions.<br><br>Incorporate all the other textual definitions and edits the reviewer asks for.	To avoid any potential noise and bias in the tree topology due to the pseudo-haploid nature of Zana's genome, low coverage and high deamination rates, we restricted the analyses to only transversions.	All "minor comments" by reviewer #3 were addressed in the updated version.

2nd Editorial Decision	21-May-2021

The manuscript is accepted for publication with the addition of the required edits.
